# Dissecting the role of putative CD81 binding regions of E2 in mediating HCV entry: Putative CD81 binding region 1 is not involved in CD81 binding

**DOI:** 10.1186/1743-422X-5-46

**Published:** 2008-03-20

**Authors:** Katharina B Rothwangl, Balaji Manicassamy, Susan L Uprichard, Lijun Rong

**Affiliations:** 1 Department of Microbiology and Immunology, College of Medicine, University of Illinois at Chicago, Chicago, IL 60612, USA; 2 Department of Medicine, College of Medicine, University of Illinois at Chicago, Chicago, IL 60612, USA; 3 Mount Sinai School of Medicine, 1 Gustave L. Levy Place, Box 1124 New York, NY 10029, USA

## Abstract

**Background:**

Hepatitis C virus (HCV) encodes two transmembrane glycoproteins E1 and E2 which form a heterodimer. E1 is believed to mediate fusion while E2 has been shown to bind cellular receptors including CD81. In this study, alanine substitutions in E2 were generated within putative CD81 binding regions to define residues critical for viral entry. The effect of each mutation was tested by challenging susceptible cell lines with mutant HCV E1E2 pseudotyped viruses generated using a lentiviral system (HCVpp). In addition to assaying infectivity, producer cell expression and HCVpp incorporation of HCV E1 and E2 proteins, CD81 binding profiles, and E1E2 association of mutants were examined.

**Results:**

Based on these characteristics, mutants either displayed wt characteristics (high infectivity [≥ 50% of wt HCVpp], CD81 binding, E1E2 expression, association, and incorporation into viral particles and proper conformation) or segregated into 4 distinct low infectivity (≤ 50% of wt HCVpp) mutant phenotypes: (I) CD81 binding deficient (despite wt E1E2 expression, incorporation and association and proper conformation); (II) CD81 binding competent, but lack of E1 detection on the viral particle, (despite adequate E1E2 expression in producer cell lysates and proper conformation); (III) CD81 binding competent, with adequate E1E2 expression, incorporation, association, and proper E2 conformation (i.e. no defect identified to explain the reduced infectivity observed); (IV) CD81 binding deficient due to disruption of E2 mutant protein conformation.

**Conclusion:**

Although most alanine substitutions within the putative CD81 binding region 1 (amino acids 474–492) displayed greatly reduced HCVpp infectivity, they retained soluble CD81 binding, proper E2 conformation, E1E2 association and incorporation into HCVpp suggesting that region 1 of E2 does not mediate binding to CD81. In contrast, conformationally correct E2 mutants (Y527 and W529) within the second putative CD81 binding region (amino acids 522–551) disrupted binding of E2 to CD81-GST, suggesting that region 2 is critical to CD81 binding. Likewise, all conformationally intact mutants within the third putative CD81 binding region (amino acids 612–619), except L615A, were important for E2 binding to CD81-GST. This region is highly conserved across genotypes, underlining its importance in mediating viral entry.

## Background

Hepatitis C virus (HCV) is a primary causative agent of chronic hepatitis. It is a positive-strand RNA virus in the family Flaviviridae that encodes a polyprotein of approximately 3,000 amino acids. This polyprotein is cleaved into ten viral proteins including two transmembrane envelope glycoproteins, E1 and E2, which are heavily N- glycosylated in their N-terminal ectodomains. Like other Flaviviruses, the interactions of the E1 and E2 glycoproteins with cell surface receptors mediate HCV entry via receptor mediated endocytosis [1]. It is believed that E1 mediates fusion of the membranes and E2 binds the cellular receptors, but it is not clear whether the fusion peptide resides in E1 or E2 [2].

Several cellular surface molecules have been implicated in HCV entry, including: CD81 [3-6], scavenger receptor class B type I (SR-BI) [7-9], the low-density lipoprotein receptor (LDLR) [10,11], Claudin-1,6 and 9 [12-14], dendritic-cell-specific intercellular adhesion molecule 3-grabbing nonintegrin (DC-SIGN) [15-17] and Liver/lymph node-specific intercellular adhesion molecule-3-grabbing integrin (L-SIGN) [18,19]. While L-SIGN and DC-SIGN are not expressed on hepatocytes, it is believed that dendritic cells expressing these molecules facilitate persistent infection by capturing and delivering the virus to the liver [18,19]. SR-BI is a multiligand receptor that binds several lipoproteins, including HDL, LDL and VLDL. It is primarily expressed in the liver and facilitates the uptake of lipids [20,21]. In infected patient's sera, HCV is found associated with LDL and VLDL, leading to the hypothesis that HCV may be "hitching a ride" with the lipoproteins to infect susceptible cells via lipoprotein receptors.

The role and requirement for CD81 in HCV entry has been thoroughly characterized and documented [3-6,22,23]. CD81 is a non-glycosylated, membrane bound protein characterized by four transmembrane domains and a small (SEL) and large (LEL) extracellular loop [24-28]. This protein is present on virtually all nucleated cells. Experiments establishing a definitive role for CD81 in HCV infection have been achieved using the retroviral pseudoparticle (HCVpp) and the recently developed *in vitro *HCV infectious clone systems [29-32]. The LEL of CD81 has been identified as the binding region of HCV E2 and critical amino acids for maintaining this interaction have been determined [33,34]. On the other hand, while several putative CD81 binding regions of HCV E2 have been identified, the critical amino acids of the E2 protein that bind CD81 are not well defined. The first proposed region spans the second hypervariable domain, extending from amino acid 474–492 [35-39]. The second region identified spans position 522–551 [35-39] and the third region is between amino acids 612–619 [35,36].

Notably, the amino acid composition of these regions varies significantly between individual viral genomes because HCV undergoes rapid genetic change requiring classification into multiple, naturally occurring genotypes. Amino acid sequences between these different genotypes vary approximately 30% and even within a single genotype, differences can range from 5–10% [40]. Thus, within HCV-infected individuals, the virus exists as a quasispecies. This is presumably due to both the random, high error rate of viral RNA polymerase as well as immune pressure [41]. Because the E2 glycoprotein varies so much, identifying conserved residues within these putative regions that are critical for maintaining the interaction between CD81 and HCV, might provide important insight not only for elucidating the molecular mechanism of viral entry, but also for developing entry inhibitors as a novel therapeutic option.

In this study, to define residues critical for viral entry, individual alanine substitutions in the three putative CD81 binding regions were generated via site-directed mutagenesis. The strategy was to target residues that are highly conserved across several strains of HCV, as retention of specific residues across genetically diverse genotypes strongly implicates those residues as being important for the interaction between HCV E2 and CD81. Although the hypervariable region II (HVR II) extends into the first putative CD81 binding region targeted (residues 474–482), residues Y474 and D481 are very highly conserved and were therefore also targeted in this study. Susceptible cell lines were challenged with HCV E1E2 pseudovirus (HCVpp) containing the individual mutations to determine the effect of each mutation on HCVpp infectivity. Additionally, producer cell expression and HCVpp incorporation of HCV E1 and E2 proteins, CD81 binding profiles, conformation and E1E2 association of mutants were also examined.

## Results

### Identification of highly conserved, charged, hydrophobic residues within the putative CD81 binding regions of E2

**Figure 1:**
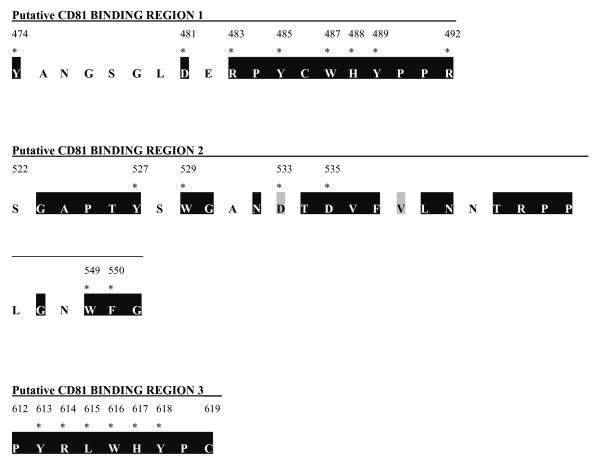
**Conserved residues within the putative CD81 binding domains of E2**. HCV strains from the Los Alamos HCV sequence database were aligned. Three regions previously implicated in CD81 binding were analyzed. Amino acids are numbered relative to the AUG start codon of the H77 strain shown and used in this study. The hyperconserved (black rectangles) targeted (asterisk) residues for alanine substitution are indicated.

Three putative CD81 interaction sites on HCV E2 have been previously identified; region 1, 474–492 [35-39]; region 2, 522–551 [35-39]; and region 3, 612–619 [35,36]. Remarkably, although E2 is subject to strong immune selective pressure *in vivo*, sequence alignment indicates that there is a high degree of sequence conservation within these three regions, consistent with the idea of these regions having functional importance. Among the three putative CD81-binding regions, region 3 (residues 612–619) is the most conserved, while region 1 (residues 474–492) has the greatest sequence variability, which is expected as the second hypervariable region (HVR II) extends into positions 474–482 (Fig. 1). Although within the HVR II, amino acids Y474 and D481 are still very highly conserved and were therefore targeted. Being interested in identifying amino acids that directly mediate HCV E2 protein-protein interactions with CD81, we decided to focus in large part on charged, hydrophobic residues conserved in these regions.

### Effect of E2 alanine substitutions on the infectivity of HCVpp

**Figure 2:**
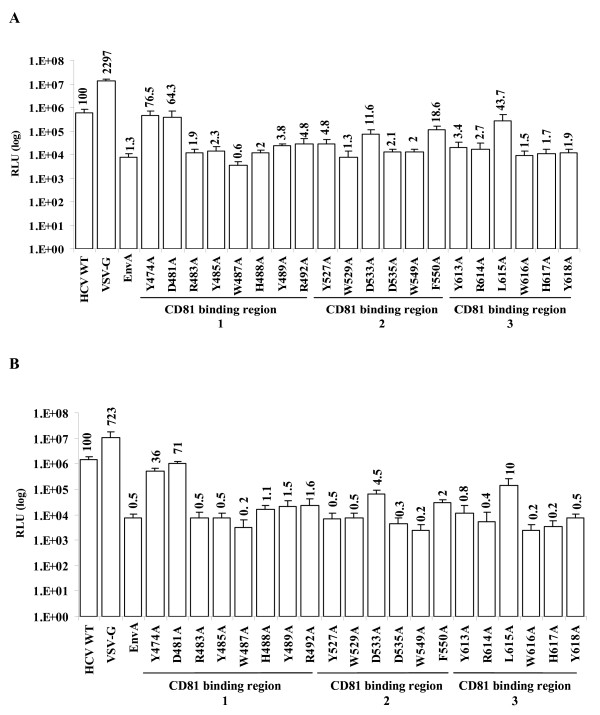
**Alanine substitutions within putative CD81 binding regions dramatically affect HCVpp entry**. 293T cells were cotransfected with the HIV-luc packaging vector along with HCV E1E2 mutant expression plasmids. HCVpp was harvested at 24 h post-transfection and used to infect susceptible cell lines (A) Huh7 and (B) Hep3B. Infectivity was measured 72 h pi using a luciferase reporter assay. Infectivity of each mutant is expressed as a percentage of the infectivity observed for the wild-type (wt) H77 HCV E1E2. Values shown are the mean and standard error for a minimum of three assays.

To identify which of the charged, conserved amino acids in the three putative CD81 interaction sites of E2 are critical for infectivity, a panel of alanine substitutions was generated within the context of H77 E2 (Fig. 1 and Fig. 2). The substitutions are numbered based on their position within the polyprotein of the H77 clone and use the one letter amino acid code to denote the amino acid present at the site prior to alanine substitution. After sequence confirmation of the alanine substitutions, HCVpp infectivity of permissive Huh7 and Hep3B cells was assessed by inoculating cells with HIV virions pseudotyped with either wt E1E2 or the mutant E1E2 glycoproteins (Fig. 2). While Huh7 cells support robust HCV infection *in vitro *[29-32] and are thus obviously a relevant cell lines for this analysis, confirmatory screening was also performed in Hep3B cells, which have been shown to be permissive for HCVpp entry. Infectivity was determined as a measure of luciferase activity. In these experiments, VSVG/HIV virions were used as a positive control. As expected, infection of the cells by VSVG/HIV virus leads to a high level of luciferase activity (~10^7 ^RLU) (Fig. 2). Infection of the target cells by wt HCV E1E2/HIV virus resulted in luciferase levels at least 2 log above a negative control, EnvA/HIV [42]. In the first putative CD81 binding region, two mutations at positions 474 and 481 retained a significant degree of infectivity relative to that of wt HCVpp E1E2 control. D481A demonstrated quantitatively similar infectivity in both Huh7 (Fig 2A) and Hep3B (Fig. 2B) cells (64% and 71% respectively), compared to wt. While mutant Y474A retained a higher percent infectivity in the Huh7 cells (76%) compared to Hep3B cells (36%), in both cell lines Y474A exhibited one of the highest levels of infectivity among the mutants tested. In contrast to these 2 mutants, the remaining alanine substitutions within the first putative CD81 binding region reduced infectivity to 5% or less of wt in both Huh7 and Hep3B cells. In the second putative CD81 binding region, alanine substitution of conserved residues severely decreased infectivity in both cell lines. While the predominant phenotype of mutations in the putative CD81 binding region 2 was ablation of infection, the D533A and F550A mutants did retain some detectable level of infectivity in Huh7 cells, 12 and 19% respectively. However this minimal level of infection was not detected in the Hep3B cell assay, confirming the severity of the infectivity defect associated with changes in these residues. Finally, in the third putative CD81 binding region, all mutations, with the exception of L615A, impaired infectivity below 4%. As seen with the Y474A mutation in region 1, the exact amount of infectivity exhibited by L615A varied between cell lines with 44% of wt levels observed in Huh7 compared to 10% of wt levels observed in Hep3B cells; however, the trend of reduced infectivity was consistent.

Notably, the infectivity trend observed for all the mutants was the same in both Huh7 and Hep3B cells, indicating the importance of these specific conserved HCV E2 residues for HCV entry in both cell types. Interestingly however, with the exception of D481A, all mutations maintained a higher level of infection in Huh7 cells compared to Hep3B, even though the baseline of HCV wt infectivity was slightly higher in Hep3B. This was particularly evident for the Y474A and L615A mutations noted above which exhibited 76% and 44% infectivity respectively in Huh7 cells compared to 36% and 10% infectivity in Hep3B cells. It remains to be determined if these quantitative differences are informative.

### Expression and incorporation of HCV E1E2 mutants

**Figure 3:**
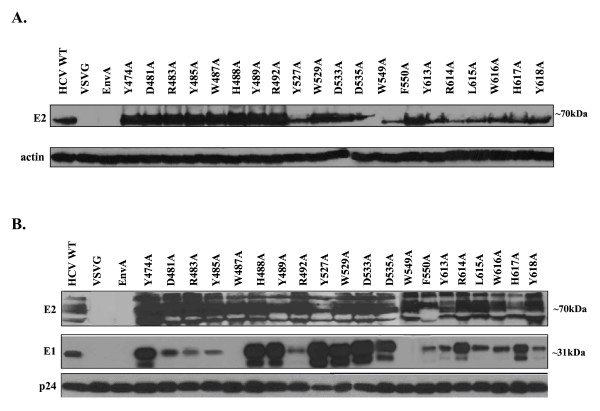
**Expression and incorporation of HCV E1E2 glycoproteins in producer cell lysate and HCVpp**. (A) 293T HCVpp producer cells were lysed and analyzed by Western Blot analysis using anti (α)-E2 and (α)-actin antibodies. Image is a composite. (B) Incorporation of HCV glycoproteins into HCVpp was determined by pelleting the virus through a 20% sucrose cushion followed by Western Blot analysis. HCV glycoproteins were identified with (α)-E2 and (α)-E1 antibodies. Detection of the HIV p24 capsid protein with an anti-HIV p24 antibody was performed as a loading control. Image is a composite.

**Figure 4:**
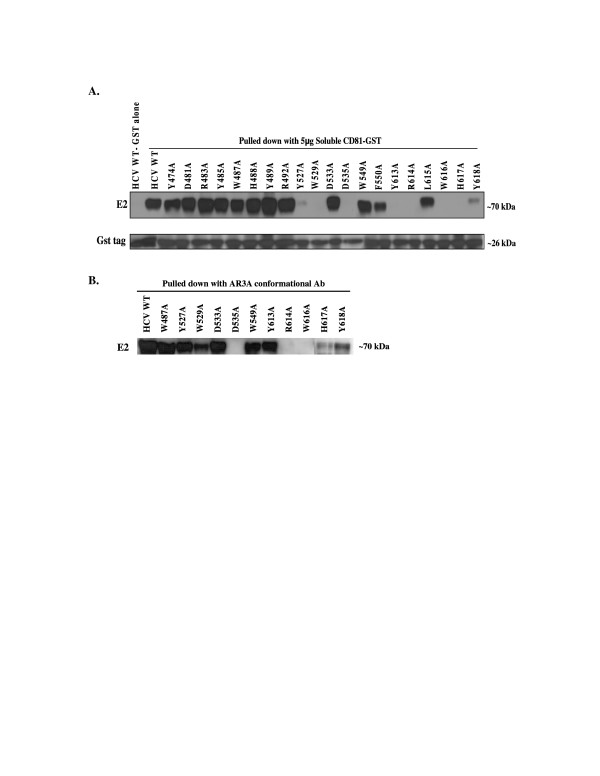
**Binding of mutant HCV E1E2 glycoproteins to soluble CD81**. (A) 293T cells transfected with HCV E1E2 wt or mutant expression vectors were lysed 24 h post-transfection. Cleared cell lysate was incubated with soluble CD81-GST fusion protein. Binding to CD81 was determined by Western Blot analysis of E2 and the GST tag. As a negative control, GST protein without soluble CD81 was incubated with HCV wt. Image is a composite. (B) 293T cells transfected with HCV E1E2 wt or specific mutant expression vectors were lysed 24 h post-transfection. Cleared cell lysate was incubated with AR3A (C1) conformational antibody to assess conformation of mutations. Immunoprecipitated proteins were detected by subsequent Western Blot analysis of E2. Image is a composite.

To confirm adequate expression of the various E2 alanine substitutions, mutant E2 protein levels in the 293T producer cell lysates transiently transfected with HIV-luc backbone and the HCV E1E2 glycoprotein plasmids were examined by Western Blot analysis. Actin levels were used as a control for protein loading. When probed with anti-E2 antibody, a band of ~70kDa was detected in the cell lysate of wt and all mutant glycoprotein transfected cells, corresponding to the size of the HCV E2 protein (Fig. 3A). Overall, cell lysate levels of E2 were reduced for the mutants in the putative CD81 binding regions 2 and 3, compared to region 1 (Fig. 3A). To determine if this reduced intracellular expression has an effect on E1E2 incorporation onto the viral particle, virions were pelleted through a 20% sucrose cushion and examined by Western Blot, probing for p24 capsid levels to control for pseudovirus particle loading. E2 extracted from the viral particle displayed a diffuse migration pattern with at least three distinct bands, most likely due to extensive N-linked glycosylation (Fig. 3B)[43]. These various forms of E2 were incorporated into particles regardless of the specific E2 mutation present and independent of the intracellular accumulation levels of the protein (Fig. 3A). Hence, the lower intracellular E2 levels detected for the mutants in regions 2 and 3 were not reflected in the amount of E2 incorporated into the viral particle. Levels of E1 detected on the various mutant viral particles however, varied greatly. Most dramatically, although E2 incorporation was not impaired, E1 was not detected in W487A or W549A mutant viral particles. This could either be due to the loss of the monoclonal antibody epitope the Western Blot was probed with or due to a lack of incorporation onto the viral particle. Based on these two E2 mutations coming down in the conformational antibody immunoprecipitation (Fig. 4B), we suspect E1 is present on HCVpp since both E1 and E2 need to be present for proper folding [44]. In any case, the level of E1 detected on the different mutant viral particles did not correlate with infectivity levels or correspond to a specific binding region. At the positions where greater levels of E1 were detected, the bands appeared as a couplet.

### Characterization of E2 mutant CD81 binding

Although E1 and mutant E2 glycoproteins were detected on viral particles except W487A and W549A, infectivity was nonetheless severely impaired in most of them. To establish if this was due to disruption of CD81 binding, as predicted based on the previous identification of these regions as putative CD81 binding domains, the binding of the mutants to recombinant soluble CD81-LEL [4] was assayed. In these experiments, binding of HCV E1E2 proteins to a purified GST tag was used as a control. Unexpectedly, twelve of the twenty mutants bound soluble CD81 at levels similar to wt, including all the mutations in the putative CD81 binding region 1 (Y474A, D481A, R483A, Y485A, W487A, H488A, Y489A, and R492A) suggesting that this region is not directly involved in binding CD81 (Fig. 4A). The fact that HCVpp infectivity was severely reduced in response to all region 1 mutants except Y474A and D481A, therefore suggests that this region likely plays another role in the viral entry process. On the other hand, while several of the mutations in regions 2 and 3 (D533A, W549A, F550A, and L615A) retained the ability to bind CD81, indicating that these specific residues are also not directly involved in the E2 and CD81 interaction, eight of the substitution mutants in these domains did not bind CD81 (Fig. 4A), consistent with the involvement of regions 2 and 3 in CD81 binding. Specifically, mutants W529A, D535A, Y613A, R614A, W616A and H617A did not bind soluble CD81 at all, and two mutants, Y527A and Y618A, exhibited dramatically reduced interaction with CD81.

To confirm that loss of CD81 binding was not due to a more general disruption of E2 structure in this region of the protein, we performed immunoprecipitation of CD81 binding deficient mutants with an antibody that recognizes a conformational epitope within the putative CD81 binding regions 2 and 3 [45]. Wt and the CD81 binding competent D533A mutants as well as W487A and W549A, for which we did not detect E1 on the viral particle, were captured with the conformational antibody, consistent with proper folding. While the Y527A, W529A, Y613A, H617A and Y618A E2 mutants were all deficient for CD81 binding, they too were recognized by the conformation-dependent antibody, indicating their conformation remained in tact. This strongly implicates these five residues as being critical for CD81 binding. In contrast, three of the mutations that did not bind CD81, (D535A, R614A and W616A) did not come down in the immunoprecipitation assay, suggesting mutations in these residues might have resulted in more global changes in E2 conformation. While loss of AR3A binding could also be due to changes in specific amino acids within the AR3A epitope, for the moment we consider these mutants as uninformative because the overall structure of the protein might be compromised.

### Analysis of E1 and E2 association

**Figure 5:**
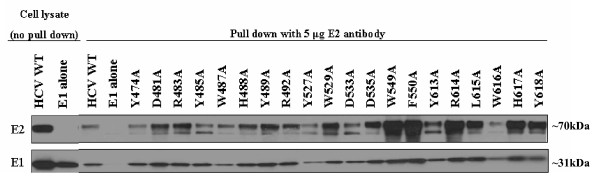
**Determining association of E1E2 mutants**. 293T cells were transfected with HCV E1E2 wt, mutant, or E1 alone glycoprotein expression plasmids. Cells were lysed and cleared cell lysate was incubated with anti (α)-E2 antibody. Immune complexes were separated by SDS-PAGE and analyzed by Western Blot for E1 to determine if the E2 and E1 glycoproteins had formed dimers. Image is a composite.

Having identified several E2 mutations that exhibit severely reduced infectivity while retaining the ability to bind CD81, we next investigated whether any of the alanine substitutions in E2 disrupted E1E2 association. It is known that E1 and E2 must properly dimerize in order to mediate HCV infectivity [2,43,46-49]. This is a relevant consideration for these mutations as E2 dimerization domains have been mapped to the transmembrane domains, a WHY motif at positions 487–489, amino acids 415–500 as well as amino acids L675, S678, L689 and L692 [48,50-53]. For this analysis, 293T cells were transiently transfected with the HCV glycoprotein constructs then lysed 48 h later. E2 protein was pulled down with polyclonal HCV E2 antibody and immunocomplexes were analyzed by Western Blot for the presence of E1 using a monoclonal antibody. To control for E2 antibody specificity, 293T cells were also transfected with E1 alone. For several mutants, most notably W549A, F550A and R614A, a greater amount of E2 was detected compared to wt (Fig. 5). Notably however, despite varying levels of E2 pulled down, E1 was detected in association with all the E2 mutants.

## Discussion

In this study we investigated the role of conserved, charged amino acid residues in three putative CD81 binding regions of HCV E2: region 1: amino acid 474–492, region 2: position 522–551 and putative region 3: amino acids 612–619 [35-39]. The 20 alanine substitution mutants characterized in this study can be classified into those displaying wt characteristics (high infectivity [≥ 50% of wt HCVpp], CD81 binding, E1E2 expression, association, and incorporation into viral particles) or segregated into 4 distinct low infectivity (≤ 50% of wt HCVpp) mutant phenotypes: (I) CD81 binding deficient (despite wt E1E2 expression, incorporation and association and proper conformation); (II) CD81 binding competent, but cannot detect E1 on the viral particle (despite adequate E1E2 expression in producer cell lysates and proper conformation); (III) CD81 binding competent, with adequate E1E2 expression, incorporation, association, and conformation (i.e. no defect identified to explain the reduced infectivity observed); (IV) uninformative mutants with potential disruptions in protein conformation (see Table 1). With only 2 mutations (W487A and W549A) appearing to result in pseudotyped particles lacking E1, most infectivity deficient alanine substitutions fell into the first or third group (i.e. CD81 binding defective or unexplained defect, respectively).

**Table 1:** 

HCVpp	Infect.	Infectivity (%)		Cellular Express.	HCVpp E1E2 Detection		E1E2 Assoc.	CD81-GST Binding	Conform.	Group
						
		Huh7	Hep3B	E2	E1	E2				
WT	+++	100	100	+++	++	+++	+	+++	+++	wt
Y474A	++	77	36	+++	+++	++++	+	+++	NA	wt&III
D481A	+++	64	71	+++	+	++++	++	+++	NA	wt
R483A	-	2	1	+++	+	++++	++	++++	good	III
Y485A	-	2	1	+++	+	++++	+	+++	good	III
W487A	-	1	0	+++	-	++++	+	+++	+++	II
H488A	-	2	1	+++	+++	++++	+	+++	good	III
Y489A	-	4	2	+++	+++	+++	++	++++	good	III
R492A	-	5	2	+++	+	++++	+	+++	NA	III
Y527A	-	5	1	+	++++	+	+	-	+++	I
W529A	-	1	1	++	++++	++++	+++	-	++	I
D533A	-	12	5	++	++++	++++	+	+++	+++	III
D535A	-	2	0	++	+++	+++	++	-	-	IV
W549A	-	2	0	+	-	++++	++++	+++	+++	II
F550A	-	19	2	++	+	+++	++++	++	good	III
Y613A	-	3	1	+	+	+++	++	-	+++	I
R614A	-	3	0	+	++	++++	++++	-	-	IV
L615A	+	44	10	+	+	++++	+++	++	NA	III
W616A	-	2	0	+	+	+++	+	-	-	IV
H617A	-	2	0	+	++	+	+++	-	+	I
Y618A	-	2	1	+	+	++++	+++	+/-	+	I

+ and - denote the properties of wt and mutants of HCV E2

"NA" indicates mutations not screened in this study and "good" indicates mutants screened in previous study [45]

Overall, our results support the notion that putative CD81 binding regions 2 and 3 are involved in CD81 binding as 5 class I mutants (Y527A, W529A, Y613A, H617A and Y618A) (see Fig. 4) demonstrate a defect in CD81 binding while maintaining proper conformation. Eleven of the twenty mutations are defective for infection (see Fig. 2), independent of CD81 binding and conformation (classes II and III) (see Table 1), suggesting that these E2 residues are involved in other essential aspects of HCV entry. All infectivity deficient mutations within the first putative CD81 binding region, with the exception of W487A, belong to the CD81 binding competent group III mutants for which we do not know why they are defective (see Fig. 2 and 4), indicating that this region, although important for HCV entry, is not directly involved in CD81 binding.

In contrast, the majority of infectivity defective substitutions in the putative CD81 binding regions 2 (Y527A, W529A and D535A) and 3 (Y613A, R614A, W616A, H617A and Y618A) demonstrate little to no CD81 binding, consistent with these regions being involved in CD81 binding. Whereas D535A, R614A and W616A all displayed a disrupted AR3A epitope in this region, indicative of more global structural aberrances Y527A, W529A, Y613A, H617A and Y618A have intact conformation and still are unable to bind CD81, defining these residues as critical for the HCV E2 and CD81 interaction. In addition, both regions 2 and 3 contain at least one group III mutation that reduces infectivity, but maintains the ability to bind CD81 (e.g. D533A, F550A, and L615A). Thus, it is likely that regions 2 and 3 are also involved in other aspects of viral entry. Notably, this is in agreement with a report by Owsianka et al. [54], which examined some of the same mutants in this region. While the degree of infectivity or CD81 binding quantified for the D533A and F550A mutants, respectively varied between the two studies, the phenotype of the mutants and conclusions drawn are qualitatively consistent

The first and third putative CD81 binding domains targeted, both contain a "WHY" motif. The first "WHY" motif (487–489) has been implicated in dimerization [52] and the second "WHY" motif (616–618) falls within region 600–620, which has been demonstrated to be involved in fusion [55]. Alanine substitution of any residues within either two of these motifs resulted in complete elimination of HCVpp infectivity. Consistent with a possible role of the region 1 "WHY" motif in proper E1E2 dimerization, Western Blot analysis of W487A HCVpp showed that substitution of tryptophan at position 487 resulted in an inability to detect the E1 epitope on HCVpp, despite the presence of E1 in producer cell lysate (see Fig. 3A). Although Lavillette et al. [55] observed a loss of both E1 and E2 glycoprotein incorporation into mutant W487A HCVpp particles, while we still detected E2 on HCVpp, our inability to detect E1 on W487A particles resulted in the classification of W487A as a group II mutant (i.e. a mutant exhibiting a defect in HCV glycoprotein incorporation), and is hence consistent with the previous report. In contrast, both H488A and Y489A mutants within region 1 did not appear to disrupt E1E2 interaction and were thus categorized as group III (i.e. no identifiable defect to explain loss of infectivity) (see Table 1).

Unlike the mutations within the region 1 "WHY" motif, CD81 binding was disrupted in all three alanine substitutions within the "WHY" motif of the third region (see Fig. 4). Mutation W616A however was not recognized in the AR3A immunoprecipitation assay, indicating that the structure of the CD81 binding epitope may have been disrupted. Therefore W616A was grouped as class IV. While lower amounts of both the H617A and Y618A mutants were captured by the AR3A antibody, suggesting that folding of these mutant proteins might be less efficient than wt, there was a population of these mutant proteins which retained this conformational epitope and could thus be analyzed for CD81 binding ability. In contrast to the H488A and Y489A mutants within region 1 however, the H617A and Y618A mutants in region 3 demonstrated reduced CD81 binding classifying then as group I mutants and implicating them as being directly involved in E2 binding to CD81.

In conclusion, we have determined that the second and third putative CD81 binding regions are responsible for mediating E2 binding CD81. In the second region, residues Y527, W529 and D535 are critical for CD81 binding. The third putative CD81 binding region comprises a CD81 binding region, as all alanine substitutions, aside from L615A, are unable to interact with CD81. This region is conserved across genotypes, underlining its significance. Finally, we have determined that the first putative CD81 binding region is not a CD81 binding region, as all mutations bind CD81 at wt levels.

## Methods

### Cell lines and antibodies

293T human embryonic kidney cells were maintained in Dulbecco's modified Eagle's media (DMEM) supplemented with 10% fetal calf serum with penicillin, streptomycin. Huh7 and Hep3B cells were maintained in DMEM supplemented with 10% fetal calf serum, penicillin, streptomycin and supplemented with 5 ml Hepes (1 M) (Gibco), and Nonessential amino acids (NEAA) (Gibco). The goat polyclonal antibody against hepatitis C virus (HCV) E2 and the monoclonal mouse antibody for E1 glycoproteins (GP) (genotype 1a) were obtained through ViroStat. The mouse anti-HIV p24 monoclonal antibody was obtained from the National Institutes of Health AIDS Research and Reference Reagent Program. Polyclonal rabbit glutathione-S-transferase (GST) antibody was obtained from NeoMarkers. The conformational anti-E2 AR3A antibody was provided by Dennis Burton, PhD from The Scripps Research Institute.

### Mutagenesis of the HCV E2 glycoprotein gene

The cDNA clone containing E1E2 from genotype 1a strain H77 in pCB6, was kindly provided by Charles Rice, PhD (Rockefeller University). All alanine substitution mutations of the HCV E2 glycoprotein were generated by site-directed mutagenesis with the Stratagene Quick-Change mutagenesis kit according to the supplier's protocols. All mutations were confirmed by DNA sequencing.

### Pseudotyping

Pseudotyped viruses were produced by cotransfecting DNA encoding wild-type (wt) or mutant glycoproteins with the Env-deficient HIV vector carrying a luciferase reporter gene (pNL4-3-Luc-R^-^-E^-^) into 293T producer cells. One microgram of the wt or mutant glycoprotein expression plasmid and 3 μg of pNL4-3-Luc-R^-^-E^- ^were used to transfect 293T cells (90% confluent) in 6-well plates with polyethylenimine (PEI). The DNA cocktail was added to 200 μl Opti-MEM media and PEI was added at 2× the volume of DNA. The mixture was incubated at room temperature for 15 min. 293T producer cells were rinsed with PBS (no Ca^++^/no Mg^++^). Eight hundred microliters of Opti-MEM was added to each well and the PEI/DNA mixture was added. After 5–6 h incubation at 37°C, the DNA cocktail was aspirated off and 3 ml cell culture media was added per well. A minimum of two wells per mutant were done at each time, for a total of 6 ml. The supernatants containing the pseudotyped viruses were collected 48 h posttransfection and filtered through a 0.45 μm-pore-size filter (Nalgene).

### Pseudotyped virus infectivity assay

Huh7 or Hep3B cells were seeded in 12-well plates at a density of 8 × 10^4 ^per well one day prior to infection. Cells were incubated with 500 μl of pseudotyped virus for 6 h, then virus was removed and cell growth media was added. The cells were lysed in 100 μl of cell culture lysis reagent (Promega) at 72 h post-infection (PI). The luciferase activity was measured with a luciferase assay kit (Promega) and a FB12 luminometer (Berthold detection system) according to supplier's protocol. Each sample was done in duplicate and experiments were repeated at least three times.

### Western Blot analysis

To determine HCV E1E2 expression and incorporation, 293T producer cells transfected with HCV E1E2/HIV plasmids as described above, were lysed in 0.5 ml of 1% Triton X-100 lysis buffer (50 mM Tris-HCl [pH 7.5], 150 mM NaCl, 5 mM EDTA) and protease inhibitor cocktail (10 μg/ml leupeptin and pepstatin, 5 μg/ml aprotinin and 2 mM phenylmethylsulfonyl fluoride) after harvesting virus and rinsing cells with PBS (no Ca^++^/no Mg^++^). The protein samples were spun down at 14 k for 10 min to clear cellular debris and transferred to fresh eppendorf tubes. SDS-PAGE loading dye was added to the protein samples, which were subsequently boiled for 5 min at 95°C, followed by sodium dodecyl sulfate-polyacrylamide gel electrophoresis (SDS-PAGE) and transferred to a polyvinyl difluoride membrane (PVDF). Membranes were then probed for HCV glycoproteins E1 and E2 and actin, p24 or GST using peroxidase-conjugated secondary antibody and chemiluminescence reagent according to the supplier's protocol (SuperSignal West pico chemiluminescent substrate, Pierce). To determine incorporation of E1 and E2 into the pseudotyped viruses, 4 ml of pseudotyped virus was layered onto a 1 ml cushion of 20% sucrose in PBS and centrifuged at 55,000 rpm for 45 min in a SW55Ti rotor (Beckman Coulter) at 16°C. The pelleted pseudovirions were lysed in 50 μl of 1% Triton X-100 lysis buffer and subjected to SDS-PAGE and Western Blot analysis.

### CD81 binding assay

The CD81 clone used was kindly provided by Shoshana Levy, PhD (Stanford University). A glutathione S-transferase (GST) fusion protein containing the large extracellular loop (LEL) of human CD81 was generated as previously described [4]. 293T producer cells were transfected with 1 μg HCV E1E2 DNA using PEI. After 48 h cells were lysed in 0.5% Triton X-100 lysis buffer with protease inhibitor on ice for 30 min. Cell lysates were clarified by centrifuging at 20,000 × g for 30 min at 4°C. Two-hundred microliters of clarified lysates from these cells were incubated with 5 μg of CD81-GST fusion protein or GST protein alone with gentle rocking at 4°C for 16 h. Fifty microliters of Glutathione Sepharose 4B (GSH) beads (GE Healthcare) rinsed three times with PBS (140 mM NaCl, 27 mM KCl, 10 mM Na_2_HPO_4_, 1.8 mM KH_2_PO_4_) were added and incubated at 4°C for 1 h. The slurry was spun down for 1 min at 14, 000 rpm and GSH beads were rinsed two times with 0.5% Triton X-100 lysis buffer. SDS-PAGE loading dye was added to the beads and samples were boiled at 95°C for 5 min. Slurry was spun down again and supernatant was collected for SDS-PAGE and Western Blot analysis.

### E2 conformational antibody immunoprecipitation

293T producer cells were transfected with 1 μg wt or a selection of CD81 binding deficient or binding competent mutant HCV E1E2 DNA constructs using PEI. After 48 h cells were lysed in 0.5% Triton X-100 lysis buffer with protease inhibitor on ice for 30 min. Cell lysates were clarified by centrifuging at 20,000 × g for 30 min at 4°C. Four-hundred microliters of clarified lysates from these cells were incubated with 1 μg of AR3A conformational antibody [45] with gentle rocking at 4°C for 16 h. Immobilized protein A (Pierce) beads were rinsed three times with PBS (140 mM NaCl, 27 mM KCl, 10 mM Na_2_HPO_4_, 1.8 mM KH_2_PO_4_). Fifty microliters of rinsed polyA beads were then added to the cell lysate/antibody cocktail and incubated with gentle rocking at 4°C for 2 h. Beads were washed three times with 100 μl 0.5% Triton lysis buffer. SDS-PAGE loading dye was added to the beads and samples were boiled at 95°C for 5 min. Slurry was spun down and supernatant was collected for SDS-PAGE separation and Western Blot analysis with a polyclonal anti E2 antibody (Virostat).

### E1E2 association pull-down

293T producer cells were transfected with 1 μg HCV E1E2 expression plasmid using PEI. Forty-eight h post-transfection cells were lysed in 4% Triton X-100 lysis buffer (4% Triton X-100, 100 mM Tris HCl [pH 8.0], 1 mM EDTA) [56] with protease inhibitor for 30 min on ice. Cell lysates were clarified by centrifuging at 20,000 × g for 30 min at 4°C. Five-hundred microliters of cell lysate was incubated with 5 μg ViroStat polyclonal, goat anti-E2 antibody for 16 h at 4°C with gentle rocking. Immobilized protein A (Pierce) beads were rinsed three times with PBS (140 mM NaCl, 27 mM KCl, 10 mM Na_2_HPO_4_, 1.8 mM KH_2_PO_4_). Fifty microliters polyA beads were then added to the cell lysate/antibody cocktail and incubated with gentle rocking at 4°C for 2 h. Beads were washed four times with 100 μl PBS with 0.2% Triton. SDS-PAGE loading dye was added to the beads and samples were boiled at 95°C for 5 min. Slurry was spun down and supernatant was collected for SDS-PAGE separation and Western Blot analysis with anti E1 antibody (Virostat).

## Authors' contributions

KBR participated in the design of the study, performed the experiments and drafted the manuscript. BM set up HIV pseudotyping system. SU participated in designing experiments and drafted the manuscript. LR designed the study and participated in drafting the manuscript.
